# Both Prelimbic and Infralimbic Noradrenergic Neurotransmissions Modulate Cardiovascular Responses to Restraint Stress in Rats

**DOI:** 10.3389/fphys.2021.700540

**Published:** 2021-08-12

**Authors:** Leandro A. Oliveira, Taciana R. S. Pollo, Elinéia A. Rosa, Josiane O. Duarte, Carlos H. Xavier, Carlos C. Crestani

**Affiliations:** ^1^Laboratory of Pharmacology, School of Pharmaceutical Sciences, São Paulo State University (UNESP), Araraquara, Brazil; ^2^Joint Federal University of São Carlos (UFSCar) - São Paulo State University (UNESP) Graduate Program in Physiological Sciences, São Carlos, Brazil; ^3^Institute of Biological Sciences, Federal University of Goiás, Goiânia, Brazil

**Keywords:** adrenoceptor, prefrontal cortex, sympathetic activity, blood pressure, heart rate, rodents, psychological stress

## Abstract

The prelimbic (PL) and infralimbic (IL) subareas of the medial prefrontal cortex (mPFC) have been implicated in physiological and behavioral responses during aversive threats. The previous studies reported the noradrenaline release within the mPFC during stressful events, and the lesions of catecholaminergic terminals in this cortical structure affected stress-evoked local neuronal activation. Nevertheless, the role of mPFC adrenoceptors on cardiovascular responses during emotional stress is unknown. Thus, we investigated the role of adrenoceptors present within the PL and IL on the increase in both arterial pressure and heart rate (HR) and on the sympathetically mediated cutaneous vasoconstriction evoked by acute restraint stress. For this, bilateral guide cannulas were implanted into either the PL or IL of male rats. All animals were also subjected to catheter implantation into the femoral artery for cardiovascular recording. The increase in both arterial pressure and HR and the decrease in the tail skin temperature as an indirect measurement of sympathetically mediated cutaneous vasoconstriction were recorded during the restraint session. We observed that the microinjection of the selective α_2_-adrenoceptor antagonist RX821002 into either the PL or IL decreased the pressor response during restraint stress. Treatment of the PL or IL with either the α_1_-adrenoceptor antagonist WB4101 or the α_2_-adrenoceptor antagonist reduced the restraint-evoked tachycardia. The drop in the tail skin temperature was decreased by PL treatment with the β-adrenoceptor antagonist propranolol and with the α_1_- or α_2_-adrenoceptor antagonists. The α_2_-adrenoceptor antagonist into the IL also decreased the skin temperature response. Our results suggest that the noradrenergic neurotransmission in both PL and IL mediates the cardiovascular responses to aversive threats.

## Introduction

The survival and adaptation of all species to aversive threats depend on a tightly controlled set of physiological responses (Sterling, 2012; McEwen, 2017). The autonomic nervous system evokes the immediate adjustments during stress that includes; for instance, the increase in arterial pressure and heart rate (HR) and the sympathetically mediated cutaneous vasoconstriction (Dampney, 2015; Crestani, 2016). The vasoconstriction of the cutaneous beds decreases the skin temperature (Vianna and Carrive, 2005; Busnardo et al., 2019). Although the relevance of the physiological responses during aversive situations, the neurobiological mechanisms involved are not completely understood.

The medial prefrontal cortex (mPFC) is activated by several aversive stimuli, such as restraint stress (Cullinan et al., 1995; Yokoyama and Sasaki, 1999; Figueiredo et al., 2002). Accordingly, the prelimbic (PL) and infralimbic (IL) subregions of the mPFC have been reported as the prominent regulators of the physiological stress responses (Ulrich-Lai and Herman, 2009; McKlveen et al., 2015, 2019; Myers, 2017). In this sense, some studies have reported that these mPFC subregions display opposite roles in the control of the responses during stressful events. For instance, studies comparing these regions identified that the PL ablation enhanced the activation of the hypothalamic–pituitary–adrenal (HPA) axis and the cardiovascular reactivity evoked by acute aversive stimuli, while the inhibition of the IL decreased these responses (Frysztak and Neafsey, 1994; Radley et al., 2006a; Tavares et al., 2009). A similar opposite role was reported for behavioral responses to stress (Vidal-Gonzalez et al., 2006). Taken together, these results indicated a region-specific regulation of responses to stress within the mPFC. Despite these fragments of evidence, the neurochemical mechanisms related to the control of stress responses by the PL and IL are poorly understood.

Noradrenergic neurotransmission is a prominent brain neurochemical mechanism involved in the regulation of stress responses (Morilak et al., 2005; Joëls and Baram, 2009; Wood and Valentino, 2017; Herman, 2018). In this sense, all adrenoceptors were identified within the mPFC (Wang et al., 2007; Ji et al., 2008; Liu et al., 2014; Santana and Artigas, 2017). Besides, the previous studies documented that the noradrenergic terminals within the mPFC arise mainly from the locus coeruleus (LC) (Sara, 2009; Chandler et al., 2013), and stress activates the LC neurons projecting to the mPFC (Borodovitsyna et al., 2020). Accordingly, aversive stimuli increased the local extracellular levels of noradrenaline within the mPFC (Nakane et al., 1994; Finlay et al., 1995; Kawahara et al., 1999; Gresch et al., 2002). The idea that the noradrenergic neurotransmission of the mPFC is a part of the central network in regulating stress responses is further supported by evidence that the lesion of catecholaminergic terminals in the mPFC inhibited the neuronal activation in other limbic structures induced by stressful stimuli (Spencer and Day, 2004). The catecholaminergic lesion within the mPFC also inhibited the activation of the HPA axis induced by restraint stress (Radley et al., 2008). Despite these fragments of evidence, the role of the noradrenergic neurotransmission of mPFC in regulating the cardiovascular responses to aversive stimuli has never been reported. Hence, this study aimed to evaluate the specific role of adrenoceptors present within the PL and IL subregions of the mPFC in cardiovascular reactivity to an acute session of restraint stress in male rats.

## Materials and Methods

### Animals

Male Wistar rats were supplied by the breeding facility of the São Paulo State University (UNESP; Botucatu, São Paulo, Brazil) and were housed in collective plastic cages (e.g., four animals/cage) in a temperature-controlled room at 24°C in the Animal Facility of the Laboratory of Pharmacology, School of Pharmaceutical Sciences, UNESP. They were kept under a 12:12-h light–dark cycle (lights on between 7:00 a.m. and 7:00 p.m.) with free access to water and standard rat chow. Housing conditions and experimental procedures were approved by the local Ethical Committee for Use of Animals (School of Pharmaceutical Sciences, UNESP), which complies with the Brazilian and international guidelines for animal use and welfare.

### Drugs and Solutions

WB4101 (selective α_1_-adrenoceptor antagonist; Tocris, Westwoods Business Park, Ellisville, MO, USA; cat. # 0946), RX821002 (selective α_2_-adrenoceptor antagonist; Tocris, cat. # 1324), propranolol (β-adrenoceptor antagonist; Sigma–Aldrich, St. Louis, Missouri, USA; cat. # P0884), tribromoethanol (Sigma–Aldrich, cat. # T48402), and urethane (Sigma–Aldrich; cat. # U2500) were dissolved in saline (NaCl 0.9%). Flunixin meglumine (Banamine, Schering Plough, Cotia, São Paulo, Brazil) and the polyantibiotic preparation of streptomycins and penicillins (Pentabiotico, Fort Dodge, Campinas, São Paulo, Brazil) were used as provided.

### Surgical Preparation

Rats underwent stereotaxic surgery at least 1 week after they arrived in the Animal Facility of the Laboratory of Pharmacology, UNESP. For this, animals were anesthetized with tribromoethanol [250 mg/kg, intraperitoneal (i.p.)], the scalp was anesthetized with 2% lidocaine, and the skull was exposed. Then, by using a stereotaxic apparatus (Stoelting, Wood Dale, IL, USA), stainless steel cannulas (i.e., 26 G, 10-mm long) directed to either the PL or IL were bilaterally implanted. The stereotaxic coordinates for the PL were as follows: anteroposterior = +3.3 mm from bregma, lateral = +1.9 mm from medial suture, and ventral = −2.4 mm from the skull. The stereotaxic coordinates for the IL were as follows: anteroposterior = +3.3 mm from bregma, lateral = +2.7 mm from medial suture, and ventral = −3.2 mm from the skull. For both structures, cannulas were implanted with an inclination of 24°. Dental cement was used to fix cannulas to the skull. After surgery, the rats were treated with a polyantibiotic preparation containing streptomycins and penicillins to prevent infection [560 mg/ml/kg, intramuscular (i.m.)] and the non-steroidal anti-inflammatory flunixin meglumine for post-operative analgesia [0.5 mg/ml/kg, subcutaneous (s.c.)].

One day before the trial, rats were again anesthetized with tribromoethanol (250 mg/kg, i.p.), and a polyethylene cannula (a 4-cm segment of PE-10 bound to a 13-cm segment of PE-50) (Clay Adams, Parsippany, NJ, USA) was implanted into the abdominal aorta through the femoral artery for cardiovascular recording. The catheter was tunneled under the skin and exteriorized on the dorsum of the animal. After the surgery, the non-steroidal anti-inflammatory flunixin meglumine was administered for post-operative analgesia (0.5 mg/ml/kg, s.c.). The animals were kept in individual cages during the post-operative period and the cardiovascular recording.

### Blood Pressure and HR Recording

The cannula inserted into the femoral artery was connected to a pressure transducer (DPT100, Utah Medical Products Inc., Midvale, UT, USA). The pulsatile arterial pressure (PAP) was recorded using an amplifier (Bridge Amp, ML224, ADInstruments, Australia) and an acquisition board (PowerLab 4/30, ML866/P, ADInstruments, Australia) that is connected to a personal computer. Mean arterial pressure (MAP) and HR values were obtained from the PAP recording.

### Tail Skin Temperature Recording

The tail skin temperature was recorded using an IR digital thermographic camera (IRI4010, InfraRed Integrated Systems Ltd., Northampton, UK). The thermographic analysis was performed using a software, and the temperature was represented by color intensity variations (Vianna and Carrive, 2005; Busnardo et al., 2019). For image analysis, the temperature was measured at five points along the tail of the animal, and the mean was calculated for each recording (Oliveira et al., 2015, 2021).

### Restraint Stress

Restraint is a commonly used stressor to investigate the behavioral and physiological responses evoked by aversive stimuli in laboratory animals (Buynitsky and Mostofsky, 2009; Campos et al., 2013; Bali and Jaggi, 2015). In this study, restraint stress consisted of introducing the animals into plastic cylindrical tubes (diameter = 6.5 cm and length = 15 cm), which were ventilated by half-inch holes that comprised ~20% of the tube. The animals were maintained for 30 min into the restraint tube (Gouveia et al., 2016; Barretto-de-Souza et al., 2018b). Each animal was submitted to a single session of stress to avoid habituation (Benini et al., 2019, 2020; Santos et al., 2020).

### Drug Microinjection

The needles (33 G, Small Parts, Miami Lakes, FL, USA) used for microinjection into the PL and IL were 1 mm longer than the guide cannulas and were connected to a 2-μL syringe (7002-KH, Hamilton Co., Reno, NV, USA) through a PE-10 tubing (Clay Adams, Parsippany, NJ, USA). The intracerebral microinjections were performed within a 5-s period, and the needle was left in the guide cannula for 1 min after the microinjection before being removed. The microinjection was performed without restraining the animals, and the drugs were administrated in a final volume of 100 nl/side (Tavares et al., 2009; Fassini et al., 2016; Brasil et al., 2018).

### Histological Determination of Microinjection Sites

At the end of each experiment, animals were anesthetized with urethane (250 mg/ml per 200-g body weight, i.p.), and 1% Evans blue dye was microinjected into the brain at the same volume of drug injection (i.e., 100 nl/side) as a marker of the microinjection site. Then, the brains were removed and post-fixed in 10% formalin solution for at least 48 h at 4°C. Later, the 40-μm-thick serial sections of the mPFC region were cut using a cryostat (CM1900, Leica, Wetzlar, Germany). The sites of injection were analyzed from the serial sections according to the study of Paxinos and Watson (1997) using a light microscope (Zeiss Axioskop 2).

### Experimental Protocol

Rats were brought to the recording room in their own cage. The recording room was temperature-controlled (24°C) and acoustically isolated. Before starting the experiment, animals were allowed at least 60 min to adapt to the room conditions, such as sound and illumination.

Each animal was connected to the cardiovascular recording system and subjected to a 30-min period of basal cardiovascular recording. Then, an independent set of rats received the bilateral microinjection into either the PL or IL of vehicle (saline, 100 nl), the selective α_1_-adrenoceptor antagonist WB4101 (10 nmol/100 nl), the selective α_2_-adrenoceptor antagonist RX821002 (10 nmol/100 nl), or the β-adrenoceptor antagonist propranolol (10 nmol/100 nl). The doses were used based on the previous studies (Fernandes et al., 2003; Resstel et al., 2005; Crestani et al., 2008). Ten minutes after the treatment, rats underwent the 30-min restraint stress session. The cardiovascular parameters (i.e., MAP and HR) were continuously recorded throughout the restraint session. The tail skin temperature was measured 10, 5, and 0 min before the restraint and at every 5 min during restraint stress.

### Data Analysis

The data were expressed as mean ± SEM. The basal values (i.e., pre-stress values) of MAP, HR, and tail skin temperature after PL or IL treatment were compared using the one-way ANOVA followed by the Bonferroni *post-hoc* test. The effects of restraint stress on the cardiovascular parameters were presented as the changes from the pre-stress values. The restraint-evoked changes were obtained by subtracting the measurements recorded during stress to the mean of values recorded for 10 min before restraint onset. The two-way ANOVA, with treatment as the main factor and time as the repeated measure, was used for the analysis of the time-course curves of the restraint-evoked changes in MAP, HR, and tail skin temperature. The results of the statistical tests with *P* < 0.05 were considered statistically significant.

## Results

**Figure 1:**
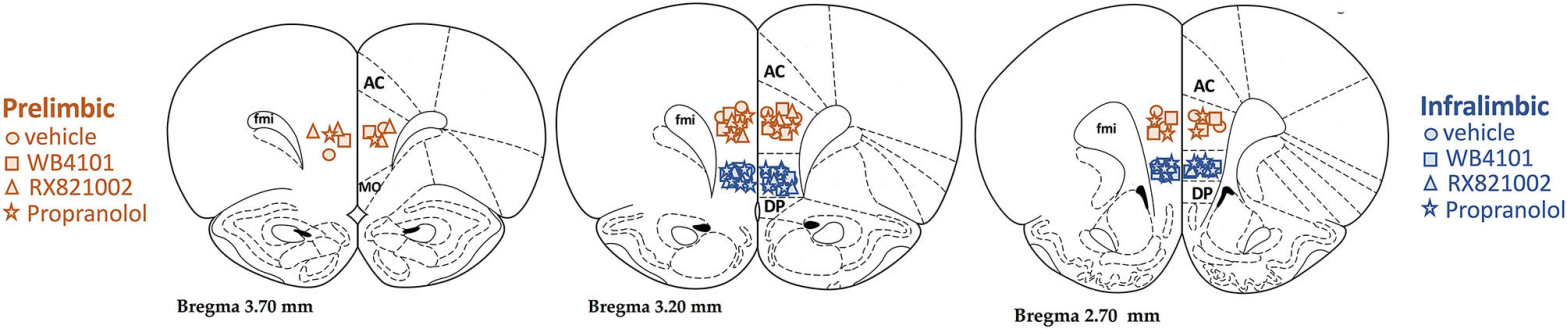
Diagrammatic representation based on the rat brain atlas of Paxinos and Watson (1997) indicating prelimbic (PL; orange symbols) and infralimbic (IL; blue symbols) microinjection sites of the selective α_1_-adrenoceptor antagonist WB4101 (squares), the selective α_2_-adrenoceptor antagonist RX821002 (triangles), the β-adrenoceptor antagonist propranolol (stars), and vehicle (circles). AC, anterior cingulate; DP, dorsal peduncular; fmi, forceps minor of the corpus callosum.

The diagrammatic representations showing the bilateral sites of microinjection into the PL and IL of all animals that were used in this study are presented in Figure 1.

### Effect of Pharmacological Treatment of PL With Adrenoceptor Antagonists in Cardiovascular and Tail Skin Temperature Changes to Acute Restraint Stress

**Figure 2:**
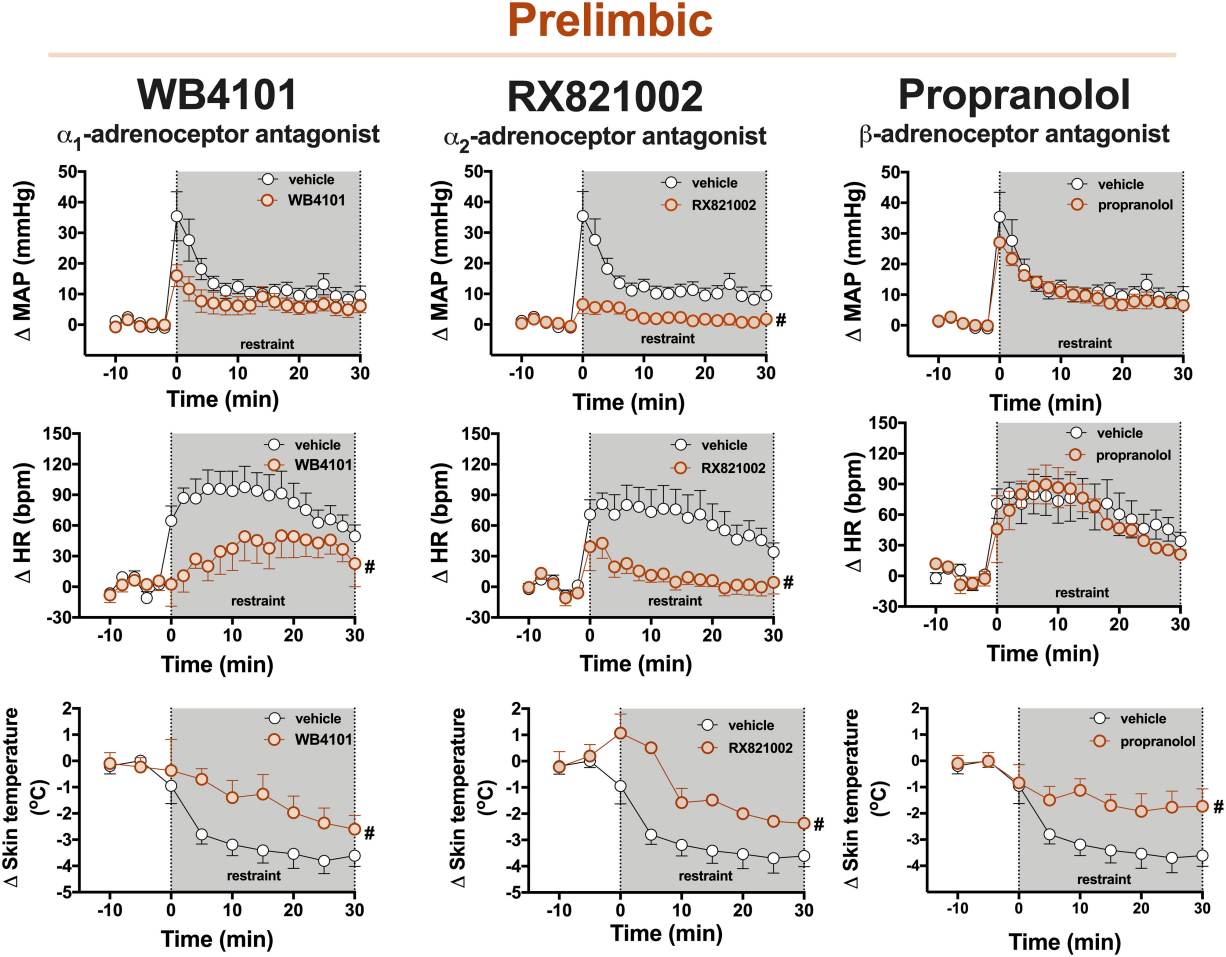
Time-course curves of the mean arterial pressure (ΔMAP), heart rate (ΔHR), and tail skin temperature (Δskin temperature) changes evoked by an acute session of restraint stress in animals treated with the selective α_1_-adrenoceptor antagonist WB4101 (10 nmol/100 nl, *n* = 6), the selective α_2_-adrenoceptor antagonist RX821002 (10 nmol/100 nl, *n* = 6), the β-adrenoceptor antagonist propranolol (10 nmol/100 nl, *n* = 6), or vehicle (100 nl, *n* = 7) into the PL cortex. Circles represent the mean ± SEM. ^#^*P* < 0.05 over the entire restraint stress period compared with the control group (vehicle), two-way ANOVA.

**α**_1_**-adrenoceptor:** The bilateral microinjection of the selective α_1_-adrenoceptor antagonist WB4101 (10 nmol/100 nl, *n* = 6) into the PL did not affect the basal values of either MAP, HR, or tail skin temperature (Table 1). Restraint stress increased MAP [*F*_(20,220)_ = 16.6, *P* < 0.0001] and HR [*F*_(20,220)_ = 9.72, *P* < 0.0001] and decreased the tail skin temperature [*F*_(8,88)_ = 15.76, *P* < 0.0001] (Figure 2). Furthermore, the microinjection of WB4101 into the PL reduced the restraint-evoked tachycardia [*F*_(1,11)_ = 5.52, *P* = 0.0406] and the drop in the tail skin temperature [*F*_(1,11)_ = 4.89, *P* = 0.0478] but without affecting the MAP response [*F*_(1,11)_ = 2.77, *P* = 0.1242] (Figure 2). The analysis also indicated a treatment–time interaction for MAP [*F*_(20,220)_ = 2.58, *P* = 0.0004] and HR [*F*_(20,220)_ = 2.35, *P* = 0.0015] but not for the tail skin temperature [*F*_(8,88)_ = 2.01, *P* = 0.0547] values.

**Table 1:** Basal parameters (i.e., pre-stress values) of the mean arterial pressure (MAP), heart rate (HR), and the tail skin temperature after pharmacological treatment of either the prelimbic (PL) or infralimbic (IL) cortex with the selective α_1_-adrenoceptor antagonist WB4101 (10 nmol/100 nl), the selective α_2_-adrenoceptor antagonist RX821002 (10 nmol/100 nl), the β-adrenoceptor antagonist propranolol (10 nmol/100 nl), or vehicle (10 nmol/100 nl).

**mPFC region/Group**	***n***	**MAP (mmHg)**	**HR (bpm)**	**Tail skin temperature (°>C)**
**PL**				
Vehicle	7	105 ± 6	365 ± 8	29.1 ± 0.6
WB4101	6	102 ± 7	378 ± 14	27.9 ± 0.4
RX821002	6	108 ± 3	370 ± 11	27.6 ± 0.4
Propranolol	6	106 ± 3	360 ± 4	27.3 ± 0.5
		*F_(3,21)_ = 0.23* *P = 0.8696*	*F_(3,21)_ = 0.54* *P = 0.6555*	*F_(3,21)_ = 2.44* *P = 0.0923*
**IL**				
Vehicle	9	107 ± 4	369 ± 8	28.1 ± 0.8
WB4101	7	105 ± 3	395 ± 11	27.9 ± 0.5
RX821002	6	108 ± 3	361 ± 3	27.5 ± 0.4
Propranolol	8	108 ± 3	361 ± 4	27.4 ± 0.3
		*F_(3,26)_ = 0.38* *P = 0.7744*	*F_(3,26)_ = 4.37* *P = 0.0129*	*F_(3,26)_ = 0.35* *P = 0.7957*

*Values are mean ± SEM. One-way ANOVA*.

**α**_2_**-adrenoceptor:** The bilateral microinjection of the selective α_2_-adrenoceptor antagonist RX821002 (10 nmol/100 nl, *n* = 6) into the PL did not affect the basal parameters of either MAP, HR, or tail skin temperature (Table 1). Restraint stress increased MAP [*F*_(20,220)_ = 12.86, *P* < 0.0001] and HR [*F*_(20,220)_ = 7.12, *P* < 0.0001] and decreased the tail skin temperature [*F*_(8,88)_ = 24.17, *P* < 0.0001] (Figure 2). The microinjection of RX821002 into the PL decreased the pressor [*F*_(1,11)_ = 11.93, *P* = 0.0054] and tachycardiac [*F*_(1,11)_ = 9.89, *P* = 0.0093] responses and the drop in the tail skin temperature [*F*_(1,11)_ = 16.68, *P* = 0.0018] evoked by restraint stress (Figure 2). The analysis also identified a treatment–time interaction for MAP [*F*_(20,220)_ = 5.89, *P* < 0.0001], HR [*F*_(20,220)_ = 2.72, *P* = 0.0002], and the tail skin temperature [*F*_(8,88)_ = 3.32, *P* = 0.0023].

**β-adrenoceptor:** The bilateral microinjection of the PL with the β-adrenoceptor antagonist propranolol (10 nmol/100 nl, *n* = 6) did not affect the baseline values of either MAP, HR, or tail skin temperature (Table 1). Restraint stress increased MAP [*F*_(20,220)_ = 24.63, *P* < 0.0001] and HR [*F*_(20,220)_ = 12.90, *P* < 0.0001] and decreased the tail skin temperature [*F*_(8,88)_ = 18.90, *P* < 0.0001] (Figure 2). Treatment of PL with propranolol decreased the drop in the tail skin temperature evoked by restraint [*F*_(1,11)_ = 5.30, *P* = 0.0420] but without affecting the pressor [*F*_(1,11)_ = 0.36, *P* = 0.5595] and tachycardiac [*F*_(1,11)_ = 0.12, *P* = 0.7293] responses (Figure 2). The analysis also indicated a treatment–time interaction for skin temperature [*F*_(8,88)_ = 2.86, *P* = 0.0072] but not for MAP [*F*_(20,220)_ = 0.66, *P* = 0.8694] and HR [*F*_(20,220)_ = 0.54, *P* = 0.9458] measurements.

### Effect of Pharmacological Treatment of IL With Adrenoceptor Antagonists in Cardiovascular and Tail Skin Temperature Changes to Acute Restraint Stress

**Figure 3:**
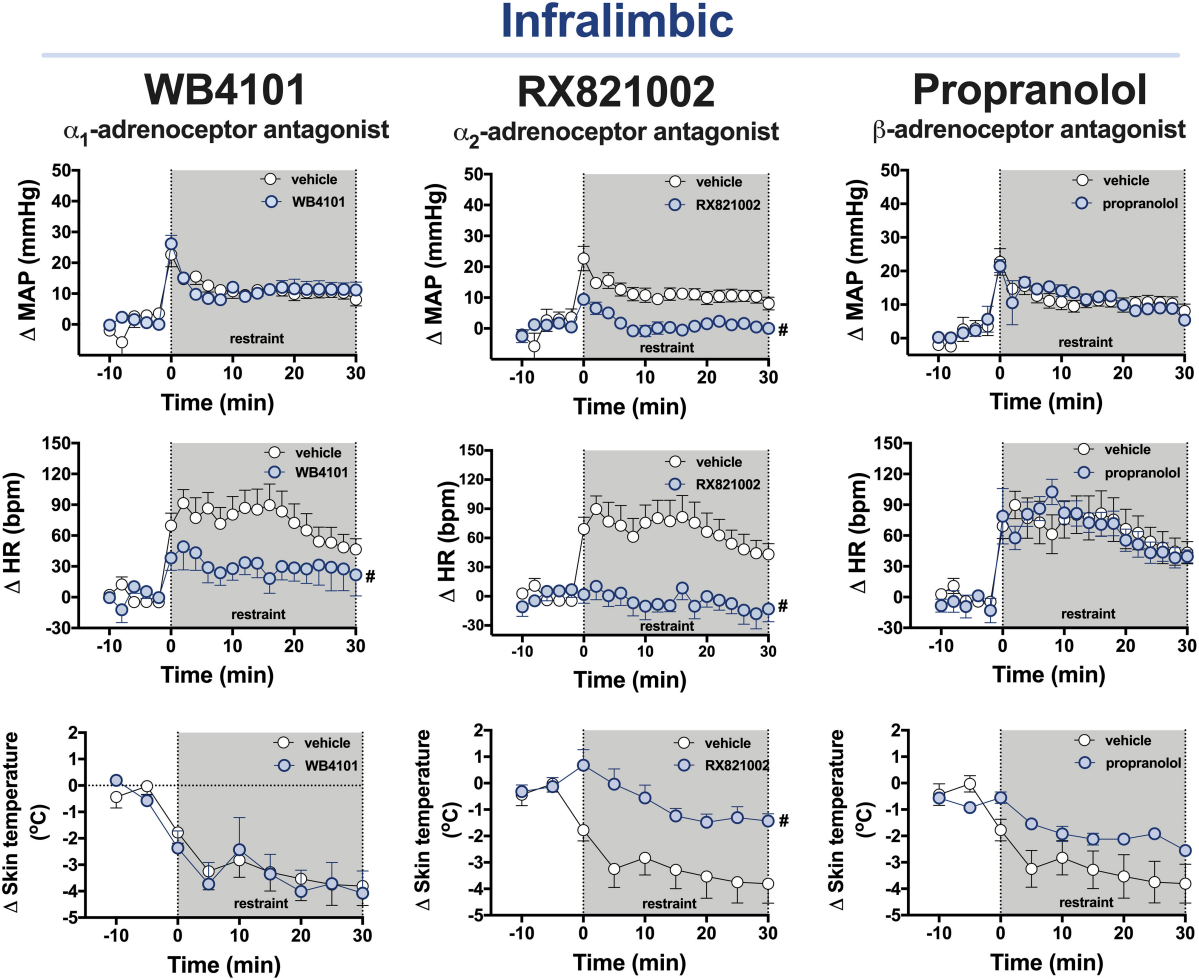
Time-course curves of the mean arterial pressure (ΔMAP), heart rate (ΔHR), and tail skin temperature (Δskin temperature) changes evoked by an acute session of restraint stress in animals treated with the selective α_1_-adrenoceptor antagonist WB4101 (10 nmol/100 nl, *n* = 7), the selective α_2_-adrenoceptor antagonist RX821002 (10 nmol/100 nl, *n* = 6), the β-adrenoceptor antagonist propranolol (10 nmol/100 nl, *n* = 8), or vehicle (100 nl, *n* = 9) into the PL cortex. Circles represent the mean ± SEM. ^#^*P* < 0.05 over the entire restraint stress period compared with the control group (vehicle), two-way ANOVA.

**α**_1_**-adrenoceptor:** The bilateral microinjection of the selective α_1_-adrenoceptor antagonist WB4101 (10 nmol/100 nl, *n* = 7) into the IL did not affect the baseline parameters of either MAP or tail skin temperature (Table 1). The one-way ANOVA indicated a significant effect of pharmacological treatments of IL for the HR values, but the *post-hoc* analysis did not reveal the differences between WB4101 and vehicle groups (Table 1). Restraint stress increased MAP [*F*_(20,280)_ = 19.68, *P* < 0.0001] and HR [*F*_(20,280)_ = 8.60, *P* < 0.0001] and reduced the tail skin temperature [*F*_(8,112)_ = 24.39, *P* < 0.0001] (Figure 3). The microinjection of WB4101 into the IL decreased the tachycardiac response evoked by restraint [*F*_(1,14)_ = 4.84, *P* = 0.0466] but without affecting the increase in MAP [*F*_(1,14)_ = 0.01, *P* = 0.9391] and the drop in the tail skin temperature [*F*_(1,14)_ = 0.03, *P* = 0.8533] (Figure 3). The analysis also indicated a treatment–time interaction for the HR values [*F*_(20,280)_ = 2.08, *P* = 0.0052] but not for MAP [*F*_(20,280)_ = 1.28, *P* = 0.1978] and the tail skin temperature [*F*_(8,112)_ = 0.53, *P* = 0.8270] measurements.

**α**_2_**-adrenoceptor:** The bilateral microinjection of the selective α_2_-adrenoceptor antagonist RX821002 (10 nmol/100 nl, *n* = 6) into the IL did not affect the basal values of either MAP or tail skin temperature (Table 1). The one-way ANOVA indicated a significant effect of pharmacological treatments of IL for the HR values, but the *post-hoc* analysis did not reveal the differences between RX821002 and vehicle groups (Table 1). Restraint enhanced MAP [*F*_(20,260)_ = 8.47, *P* < 0.0001] and HR [*F*_(20,260)_ = 4.08, *P* < 0.0001] and decreased the tail skin temperature [*F*_(8,104)_ = 13.15, *P* < 0.0001] (Figure 3). Treatment of the IL with RX821002 reduced the pressor [*F*_(1,13)_ = 19.14, *P* = 0.0008] and tachycardiac [*F*_(1,13)_ = 14.94, *P* = 0.0020] responses and the drop in the tail skin temperature [*F*_(1,13)_ = 7.52, *P* = 0.0168] (Figure 3). The analysis also indicated a treatment–time interaction for MAP [*F*_(20,260)_ = 3.27, *P* < 0.0001] and HR [*F*_(20,260)_ = 4.32, *P* < 0.0001] but not for the tail skin temperature [*F*_(8,104)_ = 4.03, *P* = 0.0003].

**β-adrenoceptor:** The bilateral microinjection of the β-adrenoceptor antagonist (10 nmol/100 nl, *n* = 8) into the IL did not affect the basal values of either MAP or the tail skin temperature (Table 1). The one-way ANOVA indicated a significant effect of pharmacological treatments of IL for the HR values, but the *post-hoc* analysis did not reveal the differences between propranolol and vehicle groups (Table 1). Restraint stress increased MAP [*F*_(20,300)_ = 17.61, *P* < 0.0001] and HR [*F*_(20,300)_ = 19.50, *P* < 0.0001] and decreased the tail skin temperature [*F*_(8,120)_ = 17.84, *P* < 0.0001] (Figure 3). The administration of propranolol into the IL did not affect the restraint-evoked pressor [*F*_(1,15)_ = 0.06, *P* = 0.8193] and tachycardiac [*F*_(1,15)_ = 0.04, *P* = 0.8441] responses and the drop in the tail skin temperature [*F*_(1,15)_ = 2.49, *P* = 0.1364] (Figure 3). The analysis indicated a treatment–time interaction for the values of tail skin temperature [*F*_(8,120)_ = 3.3, *P* = 0.0017] but not for MAP [*F*_(20,300)_ = 0.77, *P* = 0.7581] and HR [*F*_(20,300)_ = 0.88, *P* = 0.6156].

## Discussion

**Figure 4:**
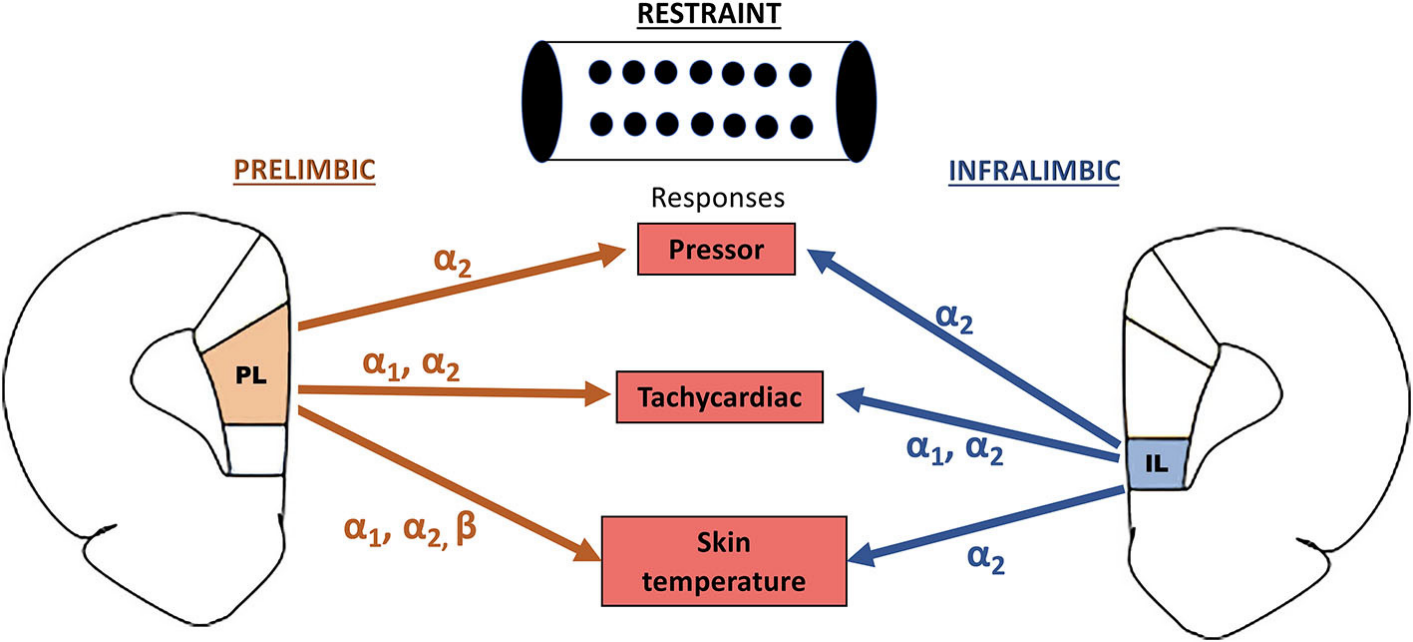
Summary of the facilitatory control of restraint-evoked pressor, tachycardiac, and skin temperature responses by α_1_-, α_2_-, and β-adrenoceptors in the PL and IL subregions of the medial prefrontal cortex.

Our results provide evidence that the noradrenergic neurotransmission in the mPFC is involved in cardiovascular adjustments during aversive threats. Despite some specific differences in the adrenoceptors involved, the data reported in this study indicate a similar facilitatory influence of the noradrenergic neurotransmissions within the PL and IL subregions of the mPFC in the restraint-evoked cardiovascular changes. Figure 4 summarizes the specific role of each adrenoceptor in the PL and IL in regulating the arterial pressure, HR, and tail skin temperature responses to acute restraint stress.

Tavares et al. (2009) reported that the non-selective synaptic inhibition caused by the local microinjection of CoCl_2_ into the PL enhanced the restraint-evoked tachycardia, whereas the decreased HR response was observed in animals subjected to the same treatment in the IL. These results indicated opposite roles of PL (inhibitory) and IL (facilitatory) in cardiac responses to restraint stress (Tavares et al., 2009). In this sense, the results reported in this study provide the first evidence that the local noradrenergic neurotransmission is involved in the facilitatory influence of the IL in restraint-evoked tachycardia. The idea that the noradrenergic neurotransmission is involved in the IL control of stress responses is supported by the previous evidence that the lesion of catecholaminergic terminals within the mPFC, including the IL region, decreased the local neuronal activation caused by stress (Spencer and Day, 2004). Nevertheless, a previous study identified that IL treatment with the excitatory amino acid, i.e., N-methyl-d-aspartate, into the IL decreased the cardiovascular responses to air-jet stress (Müller-Ribeiro et al., 2012). These results were surprising once the activation of an excitatory receptor mimicked the changes observed following the local non-selective synaptic blockade within the IL (Tavares et al., 2009). Therefore, further studies evaluating the effect of IL treatment with glutamate receptor antagonists are necessary for a full characterization of the role of glutamatergic neurotransmission in the IL control of the cardiovascular responses to stress.

As identified in this study, IL treatment with opioid receptor antagonists also decreased the blood pressure and HR responses to restraint stress (Fassini et al., 2015), thus suggesting that the noradrenergic neurotransmission might act in modulating the action of other local neurochemical mechanisms to control the cardiovascular responses during aversive threats. Although a direct interaction between noradrenergic and opioid neurotransmissions has never been reported in the mPFC, this idea is supported by evidence that α_1_- and α_2_-adrenoceptors presynaptically modulate the local release of glutamate and acetylcholine within the mPFC, respectively (Tellez et al., 1999; Luo et al., 2014). Besides, the expression of α_1_-adrenoceptor was identified in GABAergic interneurons within the IL (Santana et al., 2013). However, we cannot exclude the possibility of the direct action in pyramidal neurons, once α_1_- and α_2_-adrenoceptors were identified in these neurons and their activation increases the excitability of IL pyramidal neurons (Carr et al., 2007; Luo et al., 2014).

We identified that PL treatment with the adrenoceptor antagonists caused opposite effects in relation to that reported previously in animals treated with the non-selective synaptic blocker CoCl_2_ (Tavares et al., 2009). Similar findings were reported in relation to the control of the HPA axis response by the PL. In fact, the non-selective inhibition of the PL evoked by the local ibotenic acid lesion enhanced the restraint-evoked activation of the HPA axis (Radley et al., 2006b), whereas the ablation of noradrenergic terminals centered on the PL attenuated this neuroendocrine response (Radley et al., 2008). The attenuation of the HPA axis response to restraint evoked by the lesion of noradrenergic terminals was followed by a marked increase in the restraint-evoked activation of neurons within the PL (Radley et al., 2008). Therefore, the control of stress responses by the noradrenergic neurotransmission within the PL seems to be mediated by an inhibitory influence in the activity of local neurons. In this sense, the results reported in this study provide new evidence that this inhibitory mechanism of the noradrenergic neurotransmission within the PL is involved in the modulation of cardiovascular responses in addition to the activation of the HPA axis during aversive threats. This idea is further supported by a previous report that the microinjection of a GABA_A_ receptor antagonist within the PL decreased the restraint-evoked pressor and tachycardiac responses (Fassini et al., 2016). Therefore, the results reported in this study indicate similar roles of the noradrenergic neurotransmission (i.e., facilitatory) in the PL and IL in control of the cardiovascular responses to stress.

Evidence that α_1_-, α_2_-, and β-adrenoceptors acting post-synaptically increase the excitability of pyramidal neurons in the mPFC precludes the idea that the inhibition of pyramidal neurons by the local noradrenergic neurotransmission during aversive threats is mediated by the direct action in these cells (Carr et al., 2007; Mueller et al., 2008; Luo et al., 2014). Therefore, the control of stress responses by the noradrenergic neurotransmission in the PL seems to occur primarily *via* the modulation of other local neurochemical mechanisms. In this sense, it was reported a facilitatory influence of local GABAergic, angiotensinergic, and opioid neurotransmissions in the restraint-evoked cardiovascular responses (Fassini et al., 2014, 2016; Brasil et al., 2018). The control by α_2_-adrenoceptors might also be mediated by autoreceptors inhibiting the local noradrenaline release (Dwyer et al., 2010; Devoto et al., 2015).

Both PL and IL are connected with other limbic structures involved in the cardiovascular control during aversive threats (Hurley et al., 1991; Vertes, 2004; Myers et al., 2014; Wood et al., 2019). In this sense, a previous study identified that the lesion of catecholaminergic inputs within the mPFC decreased the stress-induced neuronal activation in the bed nucleus of the stria terminalis (BNST) (Spencer and Day, 2004). The BNST is a limbic structure that has been implicated in physiological and behavioral responses to stress (Crestani et al., 2013; Gouveia et al., 2016; Gomes-de-Souza et al., 2021). Glutamatergic pyramidal neurons of the mPFC project to the BNST (Hurley et al., 1991; Vertes, 2004; Wood et al., 2019), and recent studies indicate a facilitatory influence of glutamatergic neurotransmission within the BNST in the cardiovascular responses to restraint stress (Adami et al., 2017; Barretto-de-Souza et al., 2018a). Therefore, the facilitatory influence of the IL noradrenergic neurotransmission is possibly mediated by the activation of pyramidal neurons projecting to the BNST. Accordingly, the previous studies have reported that the IL is the main mPFC subregion projecting to the BNST (Vertes, 2004; Massi et al., 2008).

Although evidence that PL also innervates the BNST (Radley et al., 2009; Myers, 2017), the aim that the facilitatory influence of the PL noradrenergic neurotransmission in the restraint-evoked cardiovascular changes is mediated by the inhibition of local pyramidal neurons (refer to the earlier discussions) precludes the idea of an involvement of glutamatergic neurotransmission within the BNST. In this sense, the PL densely innervates other structures involved in stress responses, such as midbrain raphe nuclei (i.e., dorsal and median) and central (CeA) and basolateral (BLA) nuclei of the amygdala (Vertes, 2004; Ulrich-Lai and Herman, 2009). The previous studies indicated that the lesion of the CeA of borderline hypertensive rats reduced the increase in arterial pressure evoked by the intermittent foot shock (Sanders et al., 1994). Regarding the BLA, in addition to a role of local angiotensin-converting enzyme 2 (ACE2)/Ang-(1–7)/Mas receptor axis pathway (Oscar et al., 2015; Silva et al., 2020), the previous studies using the chemical blockade with muscimol in non-human primates and rodents have indicated a facilitatory role of this amygdaloid nucleus in the cardiovascular responses to stress (Salomé et al., 2007; Elorette et al., 2020). As for the projections to the BNST, the results indicating a facilitatory role of the CeA and BLA in the cardiovascular responses to stress do not support the hypothesis of an involvement of these amygdaloid nuclei in the control of the cardiovascular responses to stress by the PL noradrenergic neurotransmission. Regarding the raphe nuclei, although evidence of a facilitatory influence of medullary raphe nuclei in the stress-evoked cardiovascular responses (Nalivaiko et al., 2005; Vianna et al., 2008; Pham-Le et al., 2011; Ikoma et al., 2018), to the best of our knowledge, the role of dorsal and median raphe nuclei in the cardiovascular changes has never been reported. Therefore, further studies will be necessary for a discussion of the possible neural circuitry related to the control of the cardiovascular responses to stress by the PL noradrenergic neurotransmission.

## Conclusions

Our data indicate that the noradrenergic neurotransmission within the mPFC is a part of the central network in regulating the cardiovascular responses during aversive threats. Despite some differences in the specific adrenoceptor in the PL and IL in controlling each response evaluated in this study (Figure 4), the results reported in this study indicate that the noradrenergic neurotransmission in both mPFC subregions plays a facilitatory role in cardiovascular and autonomic responses during restraint stress.

## Data Availability Statement

The original contributions presented in the study are included in the article/supplementary material, further inquiries can be directed to the corresponding author/s.

## Ethics Statement

The animal study was reviewed and approved by Ethical Committee for Use of Animals (School of Pharmaceutical Sciences/UNESP).

## Author Contributions

LO: conceptualization, methodology, writing the original draft preparation, and visualization. TP and ER: conceptualization, methodology, formal analysis, investigation, and writing the review and editing. JD and CX: methodology, formal analysis, investigation, and writing the review and editing. CC: conceptualization, methodology, resources, data curation, writing the review and editing, visualization, supervision, project administration, and funding acquisition. All authors contributed to the article and approved the submitted version.

## Conflict of Interest

The authors declare that the research was conducted in the absence of any commercial or financial relationships that could be construed as a potential conflict of interest.

## Publisher's Note

All claims expressed in this article are solely those of the authors and do not necessarily represent those of their affiliated organizations, or those of the publisher, the editors and the reviewers. Any product that may be evaluated in this article, or claim that may be made by its manufacturer, is not guaranteed or endorsed by the publisher.
